# Dysregulation of NCAPG, KNL1, miR-148a-3p, miR-193b-3p, and miR-1179 may contribute to the progression of gastric cancer

**DOI:** 10.1186/s40659-018-0192-5

**Published:** 2018-11-03

**Authors:** Bin Song, Juan Du, De-feng Song, Ji-chen Ren, Ye Feng

**Affiliations:** 1 Department of Gastrointestinal and Colorectal Surgery, China-Japan Union Hospital, Jilin University, No.126, Xiantai Street, Changchun, 130033 China; 2 Internal Medicine 2, The Tumor Hospital of Jilin Province, Changchun, 130012 China

**Keywords:** Gastric cancer, Differentially expressed genes, Protein–protein interaction network, Regulatory network

## Abstract

**Background:**

Emerging evidence indicate that miRNAs play an important role on gastric cancer (GC) progression via regulating several downstream targets, but it is still partially uncovered. This study aimed to explore the molecular mechanisms of GC by comprehensive analysis of mRNAs and miRNA expression profiles.

**Methods:**

The mRNA and miRNA expression profiles of GSE79973 and GSE67354 downloaded from Gene Expression Omnibus were used to analyze the differentially expressed genes (DEGs) and DE-miRNAs among GC tissues and normal tissues. Then, targets genes of DE-miRNAs were predicted and the DE-miRNA–DEG regulatory network was constructed. Next, function enrichment analysis of the overlapped genes between the predicted DE-miRNAs targets and DEGs was performed and a protein–protein interactions network of overlapped genes was constructed. Finally, RT-PCR analysis was performed to detect the expression levels of several key DEGs and DE-miRNAs.

**Results:**

A set of 703 upregulated and 600 downregulated DEGs, as well as 8 upregulated DE-miRNAs and 27 downregulated DE-miRNAs were identified in GC tissue. hsa-miR-193b-3p and hsa-miR-148a-3p, which targeted most DEGs, were highlighted in the DE-miRNA–DEG regulatory network, as well as hsa-miR-1179, which targeted *KNL1*, was newly predicted to be associated with GC. In addition, *NCAPG*, which is targeted by miR-193b-3p, and *KNL1*, which is targeted by hsa-miR-1179, had higher degrees in the PPI network. RT-qPCR results showed that hsa-miR-148a-3p, hsa-miR-193b-3p, and hsa-miR-1179 were downregulated, and *NCAPG* and *KNL1* were upregulated in GC tissues; this is consistent with our bioinformatics-predicted results.

**Conclusions:**

The downregulation of miR-193b-3p might contribute to GC cell proliferation by mediating the upregulation of *NCAPG*; as additionally, the downregulation of miR-193b-3p might contribute to the mitotic nuclear division of GC cells by mediating the upregulation of *KNL1*.

## Background

Gastric cancer (GC) is type of common malignant tumor that originates from gastric epithelial cells [1]. Early-stage GC patients may show the symptoms of epigastric pain and weight loss [2]. In 2015, it was evaluated that GC is the second-most common cancer in China, and the incidence rate for GC is two-fold higher in men than in women (320.8 vs 157.2 per 100,000), with the mean age being more than 50 [3]. In China, early-stage gastric cancer has a relatively low diagnosis rate (< 10%) [4]. In addition, although data show that during 1984–2013, the 5-year relative survival rates for GC patients have improved from 17.8 to 20.3 to 22.9% in each decade, the clinical prognosis of GC is still poor [5]. Thus, it is a great challenge to explore novel biomarkers for the early diagnosis and effective treatment of GC.

The pathogenesis of GC is complex, involving factors such as dietary habits and environmental risks [6]. However, genetic factors are believed to be predominant factors causing GC [7]. Recent progress in researches on miRNAs and gene alterations in GC has been reported [8–10]. Emerging evidence indicates that miRNAs play an important role on the progression of GC, via the regulation of several downstream targets [11]; however, detailed information is still unavailable. Reportedly, the overexpression of miR-223 can promote GC invasion and metastasis via the regulation of the downstream tumor suppressor, EPB41L3 [11]. In addition, upregulated miRNA-194 may also promote the proliferation and migration of GC cells by activating Wnt signaling by targeting the negative Wnt regulator, SUFU [12]. Moreover, decreased levels of miR-4317, which targets ZNF322, is related to GC cell proliferation and S-G2/M transition [13].

In recent years, more and more researchers have attempted to explore the therapeutic targets of GC by microarray analysis of genes and miRNA expression profiles [14–16], and many genes like *ALDOB*, *MT1H*, and *KRT2*, as well as miRNAs such as miR-495-3p, miR-421, and miR-658 have been shown to be differentially expressed in GC tissues, compared to healthy control tissues [14–16]. However, the comprehensive regulatory mechanisms between those miRNAs and genes in GC have not yet been studied comprehensively.

In the present study, we used the data of GSE79973 mRNA and GSE67354 miRNA datasets to analyze the differentially expressed miRNAs (DE-miRNAs) and differentially expressed genes (DEGs), and predicted both the regulatory pairs between those DE-miRNAs and DEGs, and the function of those genes. Finally, we validated the expression changes of several DE-miRNAs and DEGs by real-time RT-PCR. Our study might not only provide the potential regulatory relationships between miRNAs and genes, but also identify important biomarkers for GC diagnosis and treatment.

## Results

### DEGs and DE-miRNAs analyses

Based on the aforementioned cut-off criteria, a set of 1303 DEGs were identified in GC tissue samples, compared to normal adjacent non-tumor mucosa samples, among which 703 DEGs were upregulated and 600 DEGs were downregulated. In addition, a total of 35 DE-miRNAs were screened between GC and normal adjacent non-tumor mucosa tissue samples, including 8 upregulated DE-miRNAs and 27 downregulated DE-miRNAs. The number of downregulated DE-miRNAs and upregulated DEGs were higher than the number of upregulated DE-miRNAs and downregulated DEGs.

### miRNA–miRNA network analysis

Among the 35 identified DE-miRNAs stated above, only 25 DE-miRNAs, which comprised 19 downregulated DE-miRNAs and 6 upregulated DE-miRNAs, were reported in the miRBase database. Of these, miRNA301b had no relevant targets. For the remaining 24 DE-miRNAs, a total of 2843 targets were found in the miRBase database. The top ten DE-miRNAs with more downstream targets (eg., hsa-miR-193b-3p, hsa-miR-148b-3p, and hsa-miR-193b-3p) and top ten targets regulated by more upstream miRNAs (eg., *MYC*, *CDKN1B*, and *GATA6*) are listed in Table 1, respectively.

**Table 1:** Top ten DE-miRNAs with most downstream targets and top ten genes regulated by most upstream miRNAs

miRNA	Degree	Gene	Degree
hsa-miR-193b-3p	844	MYC	6
hsa-miR-148b-3p	374	CDKN1B	6
hsa-miR-378a-5p	370	GATA6	5
hsa-miR-196a-5p	288	IGF1R	5
hsa-miR-100-5p	243	DNMT1	5
hsa-miR-148a-3p	187	STX16	5
hsa-miR-140-3p	174	HMGB1	5
hsa-miR-136-5p	147	NUFIP2	4
hsa-miR-196b-5p	136	RCC2	4
hsa-miR-99a-5p	128	FAM104A	4

**Fig. 1:**
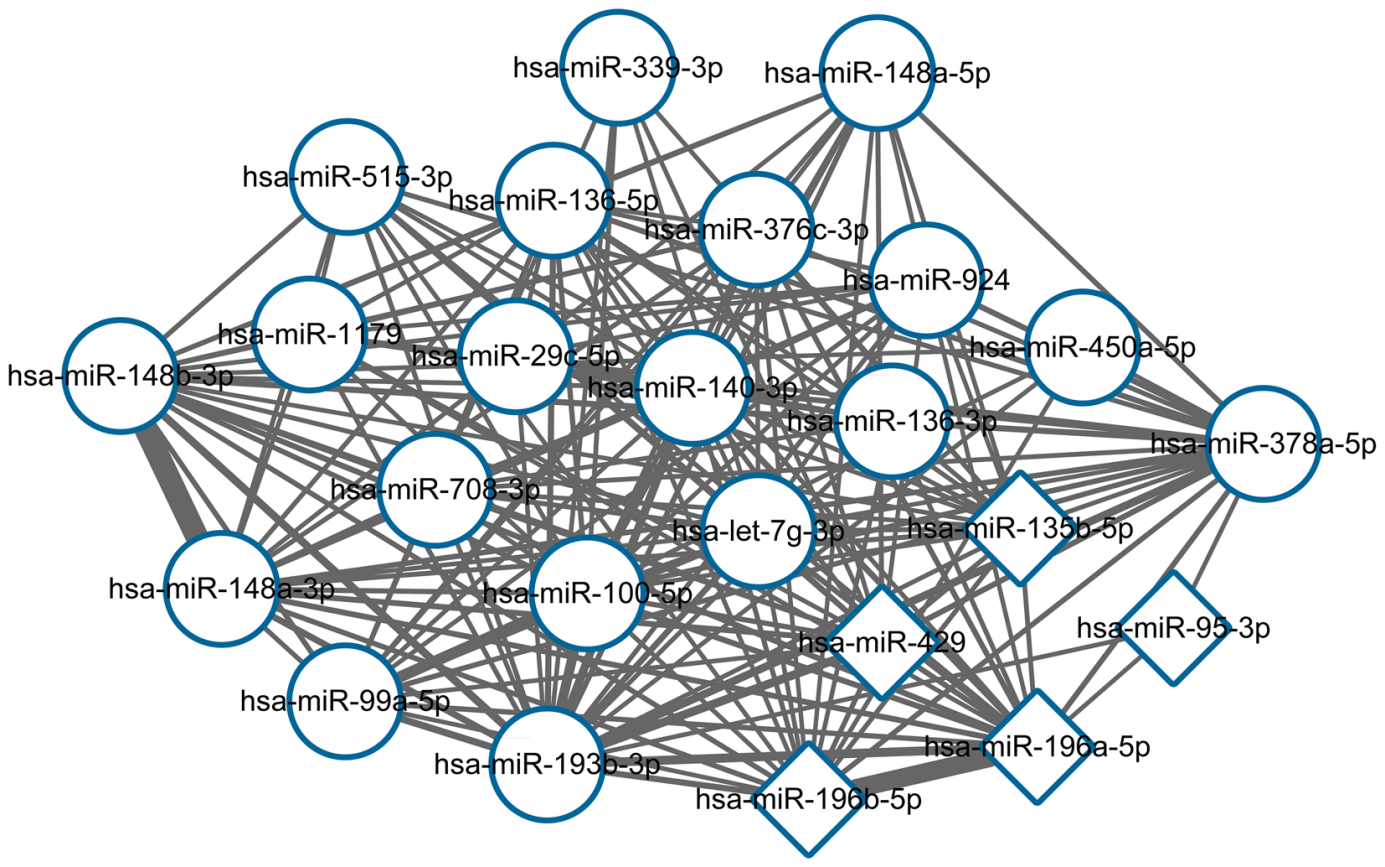
The co-regulated miRNA–miRNA target network. The circular nodes represent the downregulated miRNA, while the diamond-shaped nodes represent the upregulated miRNA. The lines indicate that the targeted genes between miRNAs are the same

The co-regulated miRNA–miRNA target network included 24 DE-miRNAs and 171 interactions (Fig. 1). The top 15 miRNA–miRNA interactions that had more number of the same target genes are listed in Table 2; this includes hsa-miR-148a-3p and hsa-miR-148b-3p, hsa-miR-196a-5p and hsa-miR-196b-5p, and hsa-miR-100-5p and hsa-miR-99a-5p.

**Table 2:** The top 15 miRNA–miRNA interactions that had more same target genes

miRNA1	miRNA2	Number of same target genes	miRNA1	miRNA2	Number of same target genes
hsa-miR-148a-3p	hsa-miR-148b-3p	113	hsa-miR-148b-3p	hsa-miR-193b-3p	21
hsa-miR-196a-5p	hsa-miR-196b-5p	88	hsa-miR-193b-3p	hsa-miR-99a-5p	16
hsa-miR-100-5p	hsa-miR-99a-5p	42	hsa-miR-148a-3p	hsa-miR-193b-3p	13
hsa-miR-100-5p	hsa-miR-193b-3p	34	hsa-miR-196a-5p	hsa-miR-140-3p	11
hsa-miR-196a-5p	hsa-miR-193b-3p	28	hsa-miR-196b-5p	hsa-miR-193b-3p	11
hsa-miR-429	hsa-miR-193b-3p	26	hsa-let-7 g-3p	hsa-miR-193b-3p	11
hsa-miR-140-3p	hsa-miR-193b-3p	24	hsa-miR-136-5p	hsa-miR-193b-3p	11
hsa-miR-193b-3p	hsa-miR-378a-5p	22			

### Pathways analysis of DE-miRNAs

**Fig. 2:**
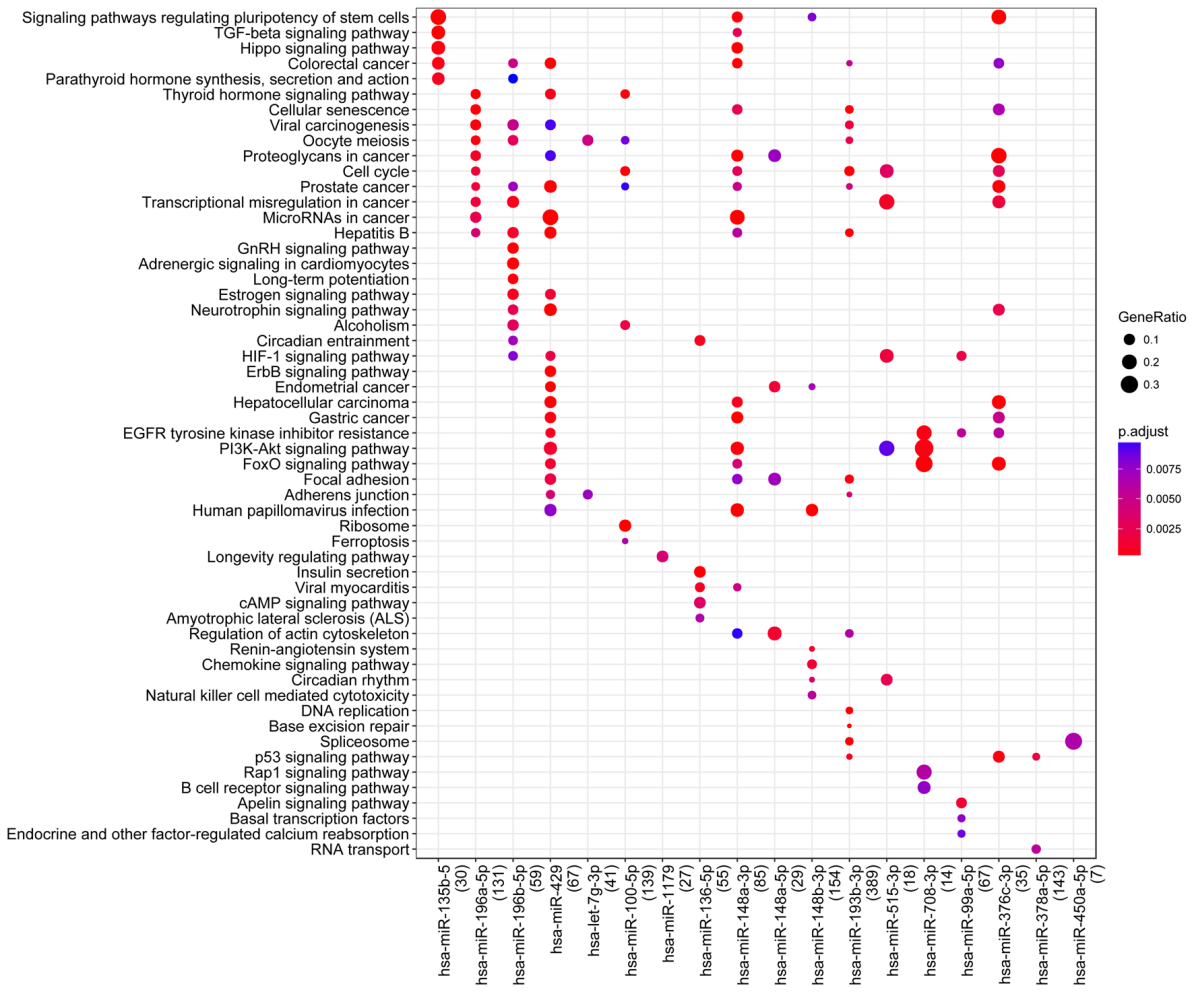
The functional enrichment result of miRNA-targeted genes. The size of each node represents the miRNA-targeted gene number ratio for the corresponding pathway, whereas the color change from blue to red indicates the p values from big to small for the corresponding pathway

In order to analysis the function of DE-miRNAs, pathways analysis based on DE-miRNA-targeted genes was performed. The results revealed that several miRNAs such as mir-196a-5p, mir-148a-3p, mir-148a-5p, mir-376c-3p, and mir-429 were closely associated with “Proteoglycans in cancer”. In addition, many miRNAs (eg., mir-100-5p, mir-148a-3p, and mir-193b-3p) were involved in the pathway of “cell cycle” (Fig. 2).

### DE-miRNA–DEG regulatory network analysis

**Fig. 3:**
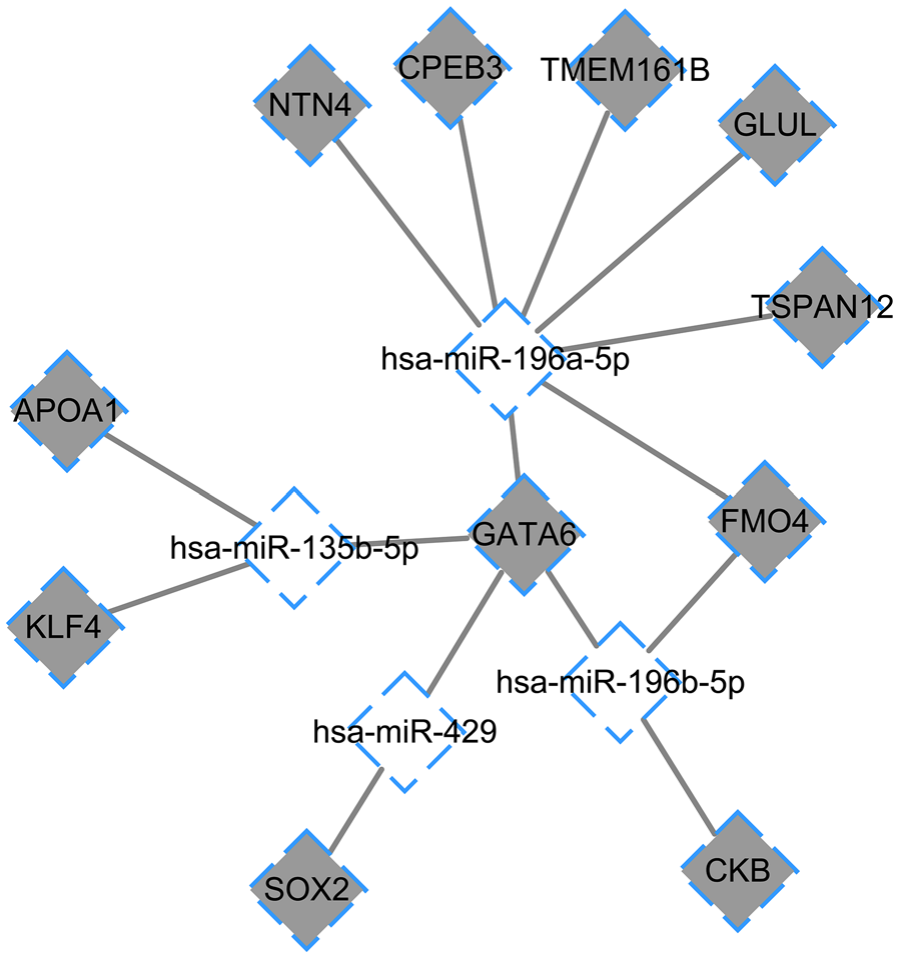
Upregulated DE-miRNA-downregulated DEG regulatory network. The white rhombus-shaped nodes represent the upregulated DE-miRNA, and the gray rhombus-shaped nodes represent the downregulated DEGs. The lines stand for the interaction between DE-miRNA and their targeted DEGs. *DEGs* differentially expressed genes

**Fig. 4:**
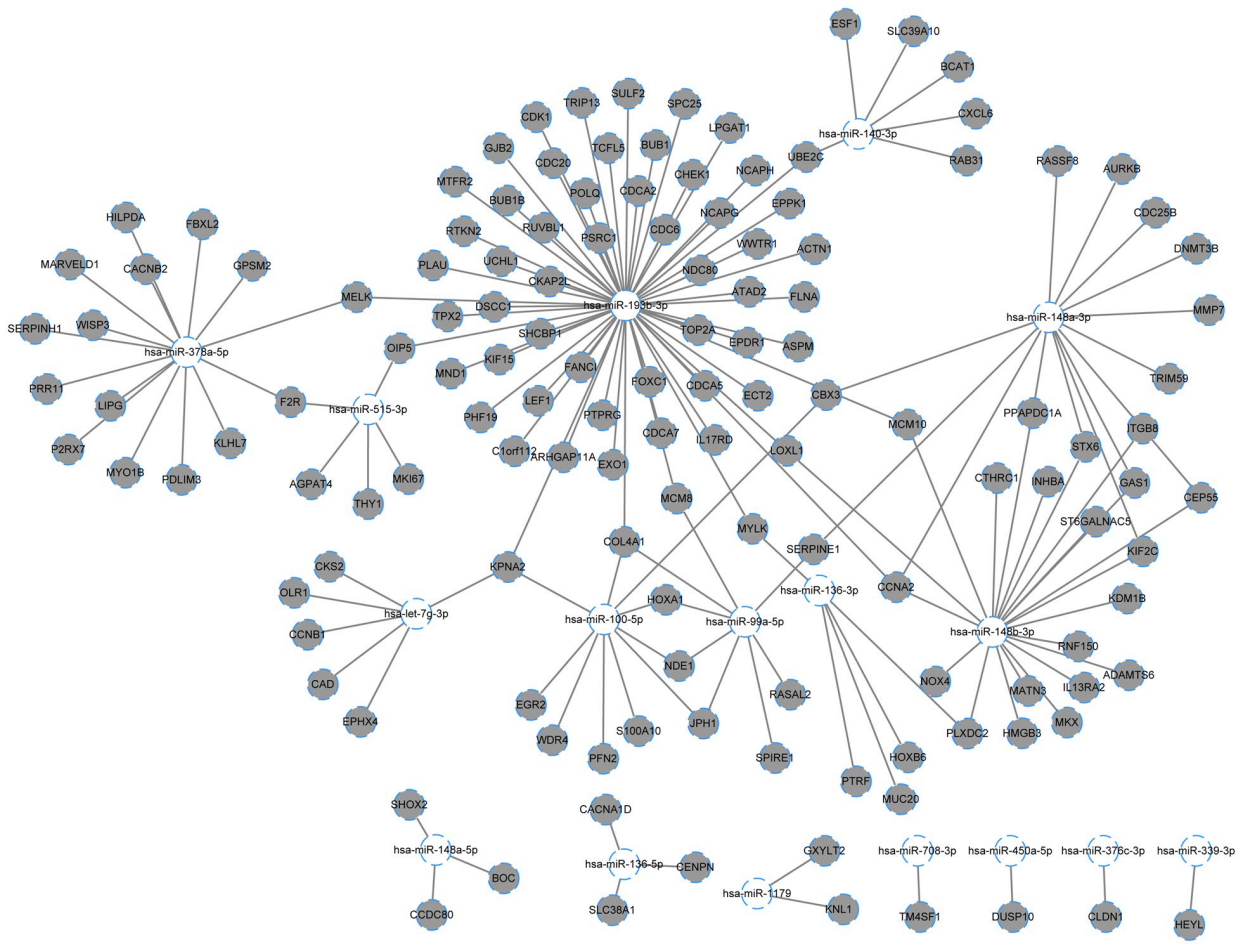
Downregulated DE-miRNA-upregulated DEG regulatory network. The white circles represent the downregulated DE-miRNA, and the gray circles represent the upregulated DEGs. The lines stand for the interaction between DE-miRNA and their targeted DEGs. *DEGs* differentially expressed genes

Based on the aforementioned methods, a total of 11 downregulated DEGs and 136 upregulated DEGs were overlapped between the targets of DE-miRNAs and all DEGs. The upregulated DE-miRNA–DEG regulatory network comprised of four upregulated DE-miRNAs, 11 downregulated DEGs, and 15 regulatory pairs, in which upregulated hsa-miR-196b-5p targeted more DEGs (eg., *GLUL* and *GATA6*) than other DE-miRNAs (Fig. 3). On the other hand, the downregulated DE-miRNA–DEG regulatory network involved 17 downregulated DE-miRNAs, 136 upregulated DEGs, and 162 regulatory pairs. In addition, hsa-miR-193b-3p and hsa-miR-148a-3p had more targeted DEGs than other miRNAs; hsa-miR-193b-3p had 59 targets (eg., *NCAPG*, *CDK1*, and *CHEK1)* and hsa-miR-148a-3p had 15 targets (eg., *KIF2C* and *MYC*) (Fig. 4).

### Functional enrichment analysis of overlapped genes

**Fig. 5:**
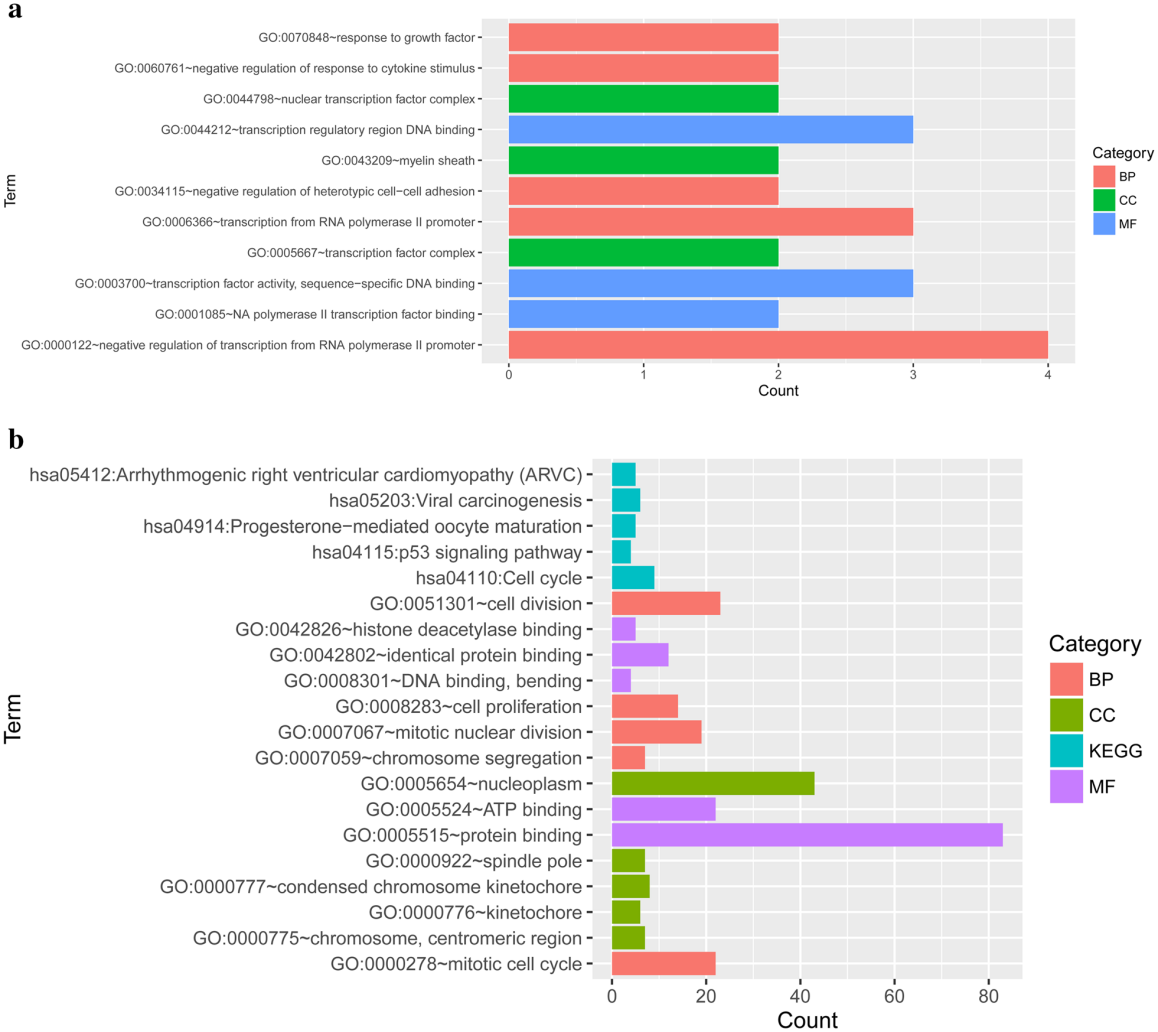
The top five enriched GO functions in the BP, CC, and MF categories, as well as the top five enriched KEGG pathways. **a** The functional enrichment results for downregulated DEGs targeted by upregulated DE-miRNAs. **b** The functional enrichment results for upregulated DEGs targeted by downregulated DE-miRNAs. *MF* molecular function, *CC* cellular component, *BP* biological process, *KEGG* Kyoto Encyclopedia of Genes and Genomes, *DEGs* differentially expressed genes

The functional enrichment analysis indicated that the downregulated DEGs were markedly related to functions such as “negative regulation of transcription from RNA polymerase II promoter” and “transcription regulatory region DNA binding’’ (Fig. 5a). However, no relevant pathways were predicted for the downregulated DEGs. In addition, most upregulated DEGs were significantly associated with functions like “cell division” (e.g., *CCNB1* and *KNL1*), “cell proliferation” (e.g., *BUB1*), and “mitotic nuclear division” (e.g., *KNL1*), and with pathways such as “p53 signaling pathway” (e.g., *CCNB1*, *CDK1*, and *CHEK1*) and “cell cycle” (e.g., *KNL1* and *NCAPG*) (Fig. 5b).

### PPI network analysis

**Fig. 6:**
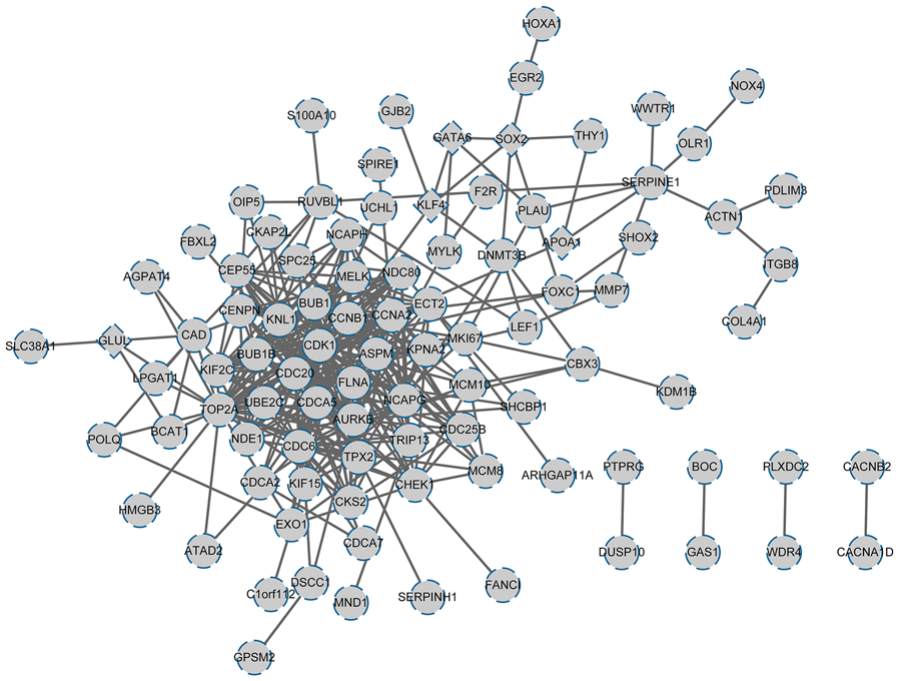
The PPI network of the overlapped DEGs. The rhombus-shaped nodes represent the downregulated DEGs, and the circular nodes represent the upregulated DEGs. The lines stand for the interactions between genes. *PPI* protein–protein interaction, *DEGs* differentially expressed genes

The interactions of the 147 aforementioned genes were investigated by constructing a PPI network (Fig. 6), which comprised of 95 nodes and 340 protein–protein interaction pairs. The top 20 nodes with high degrees are shown in Table 3, including *CDK1*, *KNL1*, *NCAPG*, and *KIF2C*.

**Table 3:** The topological property scores for nodes in the PPI network (top 20)

Node	Betweenness	Closeness	Degree
CDK1	898.30255	0.1	32
TOP2A	1276.175	0.09126214	31
AURKB	1021.4524	0.09978768	29
CDC20	342.96075	0.10010649	29
CCNB1	771.9478	0.1006424	27
BUB1	206.33615	0.1	27
CCNA2	1041.6703	0.09466264	24
BUB1B	90.71324	0.096114516	21
CDC6	251.6078	0.084761046	20
KIF2C	70.66762	0.0939061	19
CDCA5	130.50531	0.09108527	18
NDC80	67.37463	0.09680741	18
ECT2	224.30412	0.09989373	16
CASC5	213.23108	0.09447236	14
CHEK1	195.58467	0.092519686	14
NCAPG	187.03864	0.0985325	14
TPX2	195.76343	0.096114516	13
CEP55	68.46083	0.09791667	13
MELK	62.496788	0.091617934	13

*PPI* protein–protein interaction

### Validation of gene expression

**Fig. 7:**
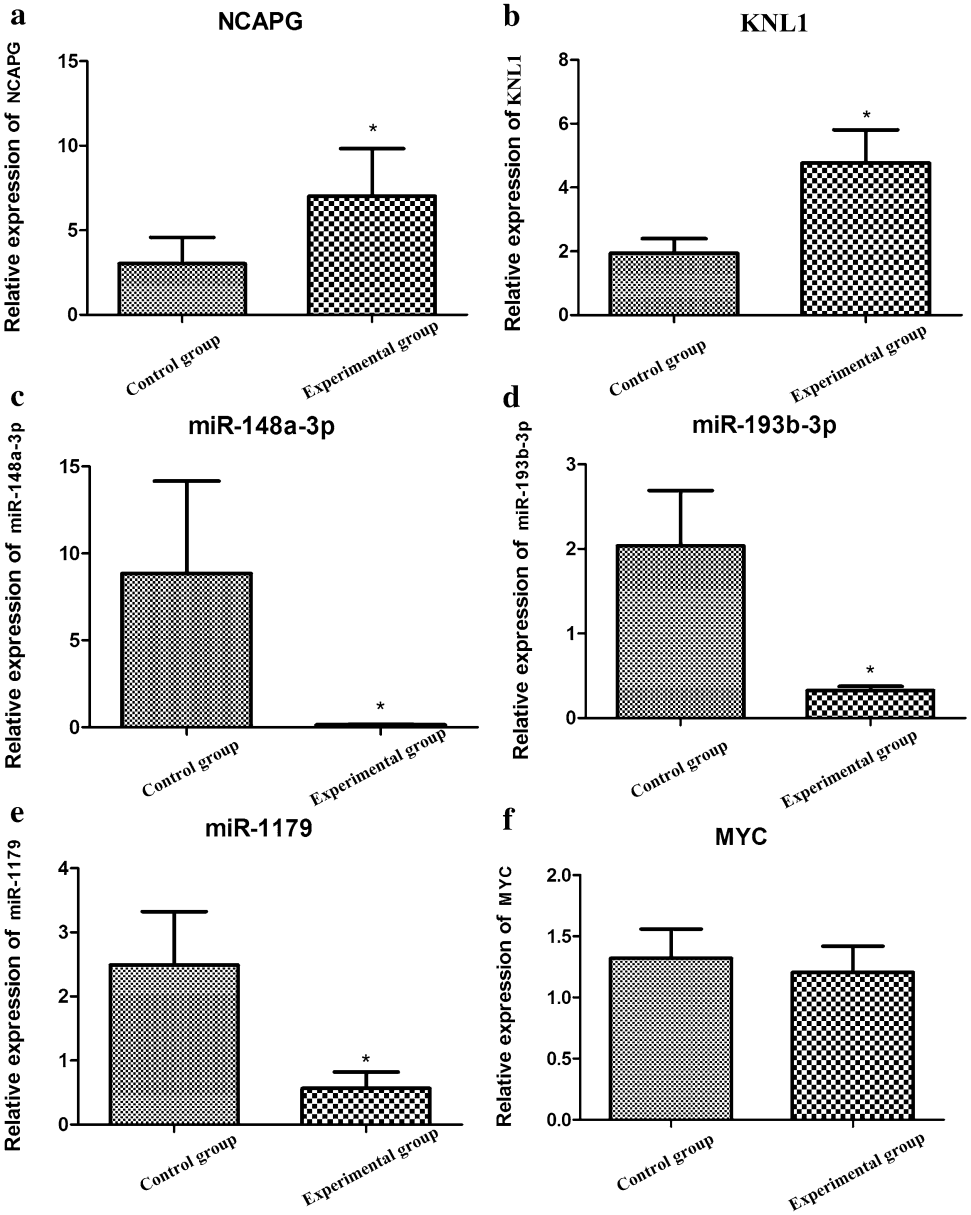
The relative miRNA expressions of *NCAPG* (**a**), *KNL1* (**b**), hsa-miR-148a-3p (**c**), hsa-miR-193b-3p (**d**), hsa-miR-1179 (**e**), and *MYC* (**f**) detected by RT-PCR in the gastric cancer tissues, compared to those in the normal controls. p < 0.05 was considered to be significantly significant

We used RT-qPCR to detect the expression of three DE-miRNAs (hsa-miR-148a-3p, hsa-miR-193b-3p, and hsa-miR-1179) and three DEGs (*MYC*, *NCAPG*, and *KNL1*). The results showed that the expression of *NCAPG* and *KNL1* were obviously increased in GC tissue samples compared to normal controls (p < 0.05, Fig. 7a, b). On the contrary, the expression of hsa-miR-148a-3p, hsa-miR-193b-3p, and hsa-miR-1179 were significantly decreased in GC tissue samples, compared to the normal controls (p < 0.05, Fig. 7c–e). Notably, those experimental results were in accordance with our bioinformatics-predicted results in the GSE79973 and GSE67354 datasets. However, no significant difference in the expression level of *MYC* was detected between the experimental and control groups (p > 0.05, Fig. 7f).

## Discussion

In our study, a total of 1303 DEGs, including 703 upregulated and 600 downregulated genes, and 35 DE-miRNAs, comprising 8 upregulated and 27 downregulated miRNAs, were identified in GC tissues, compared to the normal adjacent non-tumor mucosa tissue samples. Importantly, hsa-miR-193b-3p, which targeted 59 DEGs (eg., *NCAPG*), and hsa-miR-148a-3p, which targeted 15 DEGs (eg., *MYC*), were highlighted in the DE-miRNA–DEG regulatory network; additionally, hsa-miR-1179, which targeted *KNL1*, was newly predicted to be associated with GC. In addition, *NCAPG* and *KNL1* had higher degrees in the PPI network. Notably, overlapped DEGs were significantly associated with functions like “mitotic nuclear division” (e.g., *KNL1*) and with the pathway “cell cycle” (e.g., *KNL1* and *NCAPG*). Moreover, our RT-qPCR results showed that hsa-miR-148a-3p, hsa-miR-193b-3p, and hsa-miR-1179 were downregulated, and *NCAPG* and *KNL1* were upregulated in GC tissue, which were consistent with our bioinformatics-predicted results.

miR-148a-3p is a key regulatory factor to be involved in many cancers progression [28–30]. A study has reported that the downregulation of miR-148a-3p can promote cell migration and proliferation in patients with laryngeal squamous cell carcinoma [29]. In addition, miR-148a is detected to be downregulated in human breast cancer tissues, and its overexpression can inhibit the migration and invasion of breast cancer cells by targeting WNT-1, while inhibition of miR-148a-3p had the opposite effect [30]. Moreover, Wang et al. have suggested that the miR-148a-3p/ERBB3/AKT2/c-myc signaling axis has an important role in controlling bladder cancer progression. Consistently, we herein detected that miR-148a downregulation existed in GC tissue samples and that *MYC* was its target [28]. Furthermore, *MYC* has been suggested as a proto-oncogene; its expression is markedly high in GC tissue [31]. However, our RT-PCR results showed that there was no statistically significant difference in the expression of *MYC* between the GC tissue and control samples, and the difference with previous results might be caused due to the low number of tissue samples. Collectively, we suppose that the downregulation of miR-148a-3p might be closely associated with the development of GC, via the targeting of *MYC*.

miR-193b-3p, a tumor suppressor, is aberrantly expressed in several types of cancer. miR-193b is detected to be downregulated in ovarian cancer, and is associated with poor prognosis [32]. Similarly, Jin et al. have revealed that the reduction of miR-193b was detected in pancreatic cancer tissues and it can act as a cell-cycle brake in pancreatic cancer cells through the regulation of G1-phase arrest and fraction of cells in the S phase [33]. In addition, it has been suggested that miR-193b-3p functions as a tumor suppressor in T-cell acute lymphoblastic leukemia and can directly regulate the *MYB* oncogene [34]. Notably, our experimental results indicated that miR-193b-3p was significantly downregulated in GC tissues, and we predicted that *NCAPG*, as a target of miR-193b-3p, was upregulated and involved in the function of the cell cycle. Non-SMC Condensin I Complex Subunit G (*NCAPG*) encodes a subunit of the condensin complex I, which is associated with the proper segregation of sister chromatids in the condensation and fission of mitotic chromosomes, and is responsible for the stabilization of chromosomes during mitosis and meiosis [35]. Mitotic chromosome condensation plays a crucial role in cell proliferation and results in the reconstitution of chromosomes into rod-like mitotic chromosomes, ensuring the separation of sister chromatids during cell division. *NCAPG* has been reported to be a mitotic gene, and its overexpression is responsible for the cell proliferation and migration in hepatocellular carcinoma [36]. Consistent with our study, *NCAPG* is differentially expressed in GC tissues compared to normal control tissues, and is enriched in the cell cycle term [37]. Therefore, we speculated that the downregulation of miR-193b-3p might contribute to GC cell proliferation by mediating the upregulation of *NCAPG*.

Our study showed that miR-1179, a newly identified DE-miRNA, was related to the development of GC; RT-PCR analysis revealed that it was significantly downregulated in GC tissues. So far, there is no related report regarding the role of miR-1179 in GC. In 2017, Xu et al. had demonstrated that miR-1179 is downregulated in glioma tissues and it is associated with cell proliferation and cell cycle progression by targeting transcription factor 5 [38]. In our study, we predicted that *KNL1*, which is associated with mitotic nuclear division, is a direct target of miR-1179; experimental data showed that *KNL1* was overexpressed in GC tissues. Kinetochore Scaffold 1 (*KNL1*, also named *CASC5* or D40/AF15q14) encodes a component of the multiprotein assembly that regulates spindle assembly checkpoint and chromosome biorientation to promote accurate chromosome segregation during the cell cycle [39]. The normal expression of *KNL*1 contributes to multiple aspects of mitotic progression [40]. Evidence suggests that the overexpression of kinetochore components may lead to tumor progression by driving chromosome instability [41]. Reportedly, high D40 expression levels are detected in two human tumors cells lines: cervical cancer and lung cancer [42]. In addition, *KNL1* is involved in cell growth and division and can interact with the tumor suppressor pRb to regulate cell proliferation in cancers [42]. We suggested that as a whole, the downregulation of miR-193b-3p might contribute to the mitotic nuclear division of GC cells by mediating the upregulation of *KNL1*.

Although the study has detected the expressions of *NCAPG* and *KNL1* by RT-PCR, we fail to further validate these expressions by western blot due to no available samples and limited research funding. In future, an indepth study of regulatory mechanisms validations between genes and upstream miRNAs speculated in this study should be conducted.

## Conclusion

In conclusion, we identified a set of 703 upregulated and 600 downregulated DEGs, as well as 8 upregulated DE-miRNAs and 27 downregulated DE-miRNAs in GC tissues, in total. In addition, our results revealed that the downregulation of miR-193b-3p might contribute to GC cell proliferation by mediating the upregulation of *NCAPG*. Additionally, the downregulation of miR-193b-3p might contribute to the mitotic nuclear division of GC cells by mediating the upregulation of *KNL1*. These results provide a theoretical direction for future research with regards to the molecular mechanisms of the progression of GC.

## Methods

### Date source and data processing

The datasets GSE79973 of mRNA and GSE67354 of miRNA used in the present study were both downloaded from Gene Expression Omnibus (GEO, http://www.ncbi.nlm.nih.gov/geo/). The GSE79973 dataset was analyzed by Affymetrix Human Genome U133 Plus 2.0 Array platform, and it included 20 samples (10 GC tissue samples and 10 adjacent non-tumor mucosa samples) [14]. On the other hand, the GSE6735 dataset was analyzed by the Homo sapiens miRNA Ca_Hu_MiRNome_v2 platform. This dataset comprised data from five GC tissue samples and five adjacent non-tumor mucosa samples.

The raw data formatted as cel files and corresponding annotation files of those two datasets were obtained from GEO database. Data normalization was performed next, using the affy package (Version 1.48.0; http://www.bioconductor.org/packages/3.2/bioc/html/affy.html) in R [17], which included the background correction, quantile normalization, probe summarization, and translation of the probe ID to the gene symbol.

### Identification of differentially expressed miRNAs and genes

The T test in limma package (Version 3.26.9; http://www.bioconductor.org/packages/3.2/bioc/html/limma.html) [18] in R was used to screen the DE-miRNAs and DEGs in GC tissue samples, compared to normal adjacent non-tumor mucosa samples. The threshold values for identifying DE-miRNAs in GSE67354 were set as |log_2_ fold change (FC)| > 0.8 and p-value < 0.05, while |log_2_ FC| > 1 and p value < 0.05 were selected as the cut-off criteria for defining DEGs.

### Construction of co-regulated targets networks of DE-miRNAs

miRBase (http://www.mirbase.org/) is a public repository database, which contains all published microRNA sequences and annotation [19]. We compared the miRNA ID and mature miRNA sequence between DE-miRNAs identified above and miRNAs in miRBase database; only the DE-miRNAs with corresponding IDs in the miRBase database were reserved for following analysis. miRWalk2.0 (http://mirwalk.uni-hd.de) is a comprehensive database that provides the predicted and validated information of miRNA-target interaction [20]. The targets of DE-miRNAs were predicted from this database, and regulation pairs between the DE-miRNAs and their targeted genes were obtained. In addition, it was considered that DE-miRNAs with same target genes interacted with each other. Thus, co-regulated targets networks of miRNA–miRNA were constructed and presented using the cytoscape software (version: 3.2.0) [21].

### Functional enrichment analysis of DE-miRNAs

clusterProfiler is a package of R that applies gene classification and enrichment analyses for gene cluster comparison [22]. In our study, the pathway enrichment analysis of miRNA-targeted genes was performed using clusterProfiler package, and the miRNA-related pathway was inferred from pathways associated with their targeted genes. Next, the p value of enriched pathways was revised by BH method [23], and the revised value of p < 0.01 was chosen as the cut-off criterion for significant pathway terms.

### DE-miRNA–DEG regulatory network construction

First, we obtained the overlapped genes between the above predicted targets of DE-miRNAs and DEGs obtained from the GSE79973 dataset. Next, the overlapped upregulated genes and downregulated genes were divided. By acquiring the regulatory relationships between the upregulated DE-miRNAs and downregulated DEGs, and downregulated DE-miRNAs and upregulated DEGs, the upregulated DE-miRNAs-targeted DEG and downregulated DE-miRNAs-targeted DEG networks were constructed using the cytoscape software.

### Functional enrichment analysis of overlapped genes

Gene ontology (GO) database offers functional annotations of genes from three aspects including biological process, molecular function, and cellular component [24]. The Kyoto Encyclopedia of Genes and Genomes (KEGG) is also an important database for genome annotation, which defines the functions of genes or proteins in several specific metabolic and regulatory pathways [25]. In the present study, the GO term and KEGG pathways analysis of overlapped genes was performed by using the biocloudservice platform (http://www.biocloudservice.com/). The cut-off criterion for significant GO terms and KEGG pathways was set as p < 0.05.

### Protein–protein interactions (PPIs) network construction

The Search Tool for the Retrieval of Interacting Genes (STRING, https://string-db.org/) database provides the functional associations between proteins for more than 200 organisms [26]. In our study, the PPIs of overlapped DEGs were analyzed using STRING. Then, the obtained PPI pairs were used to construct the PPI network, which was visualized using Cytoscape [21]. The topological properties of each node in the PPI network were also analyzed.

### Validation of gene expression by real-time RT-PCR analysis

RT-PCR analysis was performed to detect the expression levels of several key DEGs and DE-miRNAs that were predicted to be closely associated with GC. The experimental material, which was five normal gastric mucosa (Normal group) and five GC tissue samples (Experimental group), were collected from five gastric cancer patients, who had undergone radical gastrectomy at the Sino-Japanese Friendship Hospital of Jilin University. All the patients had signed the informed consent before participating in the study. The study has been approved by the Ethics Committee of the Sino-Japanese Friendship Hospital of Jilin University. Total RNA was extracted using the TriZol reagent (TAKARA, Dalian, China, Cat. No. 9109), and RNA was reversed to produce complementary DNA with 5× primeScript RT Master MIX (Takara, Dalian, China, Cat. No. RR036A). After cDNA synthesis, quantitative real-time PCR was conducted with Power SYBR Green PCR Master (Thermo Scientific, Waltham, MA, USA, Cat. No. 4367659). GAPDH and U6 were selected as reference genes for quantitating DEGs and miRNAs, respectively. The primer sequences of detected genes are listed in Table 4, and the relative expression of genes was calculated using the 2^−ΔΔCt^ method [27]. All the experiments were repeated thrice.

**Table 4:** The primer sequence for each validated gene

Primer name	Primer sequence (5′-3′)
U6-F	CTCGCTTCGGCAGCACA
U6-R	AACGCTTCACGAATTTGCGT
Human-U6-RT	GTCGTATCCAGTGCAGGGTCCGAGGTATTCGCACTGGATACGACAAAATATG
GAPDH-F	TGACAACTTTGGTATCGTGGAAGG
GAPDH-R	AGGCAGGGATGATGTTCTGGAGAG
MYC-F	CCTGGTGCTCCATGAGGAGAC
MYC-R	CAGACTCTGACCTTTTGCCAGG
NCAPG-F	TTAAGGAGGCCTTTCGGCTG
NCAPG-R	TCCACAGCTGGTTCACGTTT
CASC5-F	AGAAATGGAAGAAACAGAAACAGG
CASC5-R	TGCATGTTTCCTTTCACGGG
hsa-miR-148a-3p-RT	GTCGTATCCAGTGCAGGGTCCGAGGTATTCGCACTGGATACGACACAAAG
JH-hsa-miR-148a-3p-F	GCGCTCAGTGCACTACAGAA
hsa-miR-193b-3p-RT	GTCGTATCCAGTGCAGGGTCCGAGGTATTCGCACTGGATACGACAGCGGG
JH-hsa-miR-193b-3p-F	GCGCAACTGGCCCTCAAAGT
hsa-miR-1179-RT	GTCGTATCCAGTGCAGGGTCCGAGGTATTCGCACTGGATACGACCAACCA
JH-hsa-miR-1179-F	GCGCGCAAGCATTCTTTCAT

### Statistical analysis

All results are presented as the mean ± standard error of mean (SEM). Statistical analysis of differences between groups was performed using the SPSS 22.0 software, and p < 0.05 was considered to be significant. The graph software used was Graphpad prism 5 (Graphpad Software, San Diego, CA).

## Abbreviations

GC: gastric cancer
DE-miRNAs: differentially expressed miRNAs
DEGs: differentially expressed genes
GEO: Gene Expression Omnibus
FC: fold change
GO: gene ontology
KEGG: Kyoto Encyclopedia of Genes and Genomes
PPIs: protein–protein interactions
STRING: Search Tool for the Retrieval of Interacting Genes
SEM: mean ± standard error of mean
NCAPG: Non-SMC Condensin I Complex Subunit G
KNL1: kinetochore scaffold 1
